# Epithelial differentiation of human adipose-derived stem cells (hASCs) undergoing three-dimensional (3D) cultivation with collagen sponge scaffold (CSS) via an indirect co-culture strategy

**DOI:** 10.1186/s13287-020-01645-3

**Published:** 2020-03-31

**Authors:** Minxiong Li, Jun Ma, Yanbin Gao, Mengru Dong, Zijun Zheng, Yuchen Li, Rongwei Tan, Zhending She, Lei Yang

**Affiliations:** 1 Department of Burns, Nanfang Hospital, Southern Medical University, Jingxi Street, Baiyun District, Guangzhou, 510515 People’s Republic of China; 2 Guangdong Engineering Research Center of Implantable Medical Polymer, Shenzhen Lando Biomaterials Co., Ltd., Shenzhen, 518107 People’s Republic of China

**Keywords:** Adipose-derived stem cell, Keratinocyte, Collagen sponge scaffold, Three-dimensional cultivation, Wound healing, Regenerative medicine

## Abstract

**Background:**

Three-dimensional (3D) cultivation with biomaterials was proposed to facilitate stem cell epithelial differentiation for wound healing. However, whether human adipose-derived stem cells (hASCs) on collagen sponge scaffold (CSS) better differentiate to keratinocytes remains unclear.

**Methods:**

3D cultivation with CSS on hASC epidermal differentiation co-cultured with HaCaT cells at air-liquid interface (ALI) was compared with two-dimensional (2D) form and cultivation without “co-culture” or “ALI.” Cellular morphology, cell adhesion, and growth condition were evaluated, followed by the protein and gene expression of keratin 14 (K14, keratinocyte specific marker).

**Results:**

Typical cobblestone morphology of keratinocytes was remarkably observed in co-cultured hASCs at ALI, but those seeded on the CSS exhibited more keratinocyte-like cells under an invert microscope and scanning electron microscope. Desired cell adhesion and proliferation were confirmed in 3D differentiation groups by rhodamine-labeled phalloidin staining, consistent with H&E staining. Compared with those cultured in 2D culture system or without “ALI,” immunofluorescence staining and gene expression analysis revealed hASCs co-cultured over CSS expressed K14 at higher levels at day 15.

**Conclusions:**

CSS is positive to promote epithelial differentiation of hASCs, which will foster a deeper understanding of artificial dermis in skin wound healing and regeneration.

## Background

Skin wounds resulting from various disorders (like thermal injury, trauma and chronic ulcerations secondary to diabetes mellitus, and venous stasis) [1–3], accompanied by increasing morbidity and rising risk of amputations, have caused a heavy socioeconomic burden and become a worldwide health and economic problem [4, 5]. Wound healing is a complex multi-step process that mainly experiences four continuous phases, namely hemostasis, inflammation, proliferation, and maturation or remodeling [6, 7]. And many cell types, growth factors, cytokines, and extracellular matrix (ECM) components are involved in wound healing [8, 9].

Skin re-epithelialization is a crucial step in the proliferation phase [10, 11], of which keratinocytes, the main components of the epidermis of the skin, play an important role in promoting re-epithelialization [4, 11]. Under favorable conditions, the keratinocytes from the edges of the wound begin to migrate towards the center within hours after injury and proliferate continuously until the epithelial surface is intact [12]. However, in wounds by large severe burns and severe trauma, residual keratinocytes often have defects with poor quality and insufficient quantity, which may lead to delayed wound healing or non-healing chronic wounds [13, 14]. Furthermore, keratinocytes require specific culture conditions and easily become senescent after only 10–20 doublings [15], which hampers the production of sufficient cells for graft [16].

Adipose-derived stem cells (ASCs), a type of mesenchymal stem cells (MSCs) [17–19], are adult stem cells capable of self-proliferation and multipotent potential (endoderm, mesoderm, and ectoderm) [1, 17, 20]. In particular, many researches have reported that ASCs can be transdifferentiated into keratinocytes under certain conditions [21–24]. Moreover, human ASCs (hASCs) have the advantages of wide sources, convenient access strategies, less damage to patients, rich stem cells in adipose tissue, and low immunogenicity [1, 25], indicating that hASCs transdifferentiated into keratinocytes can be a promising strategy to promote wound healing. The differentiation of stem cells depends on the microenvironment of cell growth, named “niche” [21, 26, 27]. Currently, the main strategy for the differentiation of ASCs into keratinocytes is to add relevant inducing factors to the culture medium [22, 23, 28] or co-culture with target cells [29]. Recent studies have shown a better differentiation into keratinocytes when ASCs were lifted to “air-liquid interface (ALI)” by means of co-cultivation [16]. That is, the morphology of ASCs changed from long spindles to typical cobblestone morphology, and immunofluorescence demonstrated that keratinocytes markers (such as keratin 14 and K14) became positive [30–32]. Most studies are based solely on two-dimensional (2D) differentiation model without the induction of ECM [33, 34] and increasing researches on three-dimensional (3D) cultivation using scaffolds or other biomaterials may address this issue [22, 23, 29].

With the development of tissue engineering technology, many skin scaffolds (termed as skin substitutes or artificial dermis) have been conceived as skin wound dressings [6] and applied clinically for eventual scar-free wound healing, including Pelnac® (Japan) [10], Integra® (USA) [10], and Lando® (China) [35] [36, 37]. Currently, artificial dermis commercially available mainly consists of collagen sponge scaffolds (CSSs) with multiple desirable characteristics (such as biocompatibility, an interconnected pore structure, and sufficient biodegradability) [10, 38, 39], which are utilized as templates to promote fibroblasts and endothelial cells to proliferate [40], migrate and mature into scaffolds, and promote dermal regeneration and neovascularization, significantly reducing contracture and scar formation [41–43].

Despite these, due to the fact that there is currently no product available that contains complete dermis and epidermis, autologous skin grafts in the second phase are usually required in the clinical application of artificial dermis [44]. Furthermore, 3D cultivation was reported to enhance the differentiation into keratinocyte-like cells when ASCs were cultured on components of ECM such as type IV collagen or fibronectin [1, 16], boosting it necessary to clarify the differentiation of MSCs over the existing artificial dermis.

In this study, 3D co-culture model with CSS was constructed, based on the indirect co-culture inducing principles with filter well inserts [1], to investigate the effects of CSS on the expression of keratinocytes markers in hASCs at the ALI, and the initial mechanism that might be involved was also analyzed.

## Methods

### Harvesting and cultivation of hASCs and keratinocytes

hASCs harvested from female abdominal liposuction was kindly donated by Jing Xia, Department of Plastic and Cosmetic Surgery, Nanfang Hospital. hASCs cells were cultivated in Dulbecco’s modified Eagle’s medium (DMEM, REF# C11995500BT, Gibco) with 10% fetal bovine serum (FBS, REF# 10091148, Gibco) and 1% antibiotics (penicillin and streptomycin, REF# 15140122, Gibco) at 37 °C in a humidified air with 5% CO_2_. And 3rd–5th passages (P3–P5) of hASCs have been used for all experiments.

As a substitute for keratinocytes in scientific research [45], HaCaT cells were kindly donated by Jinmei He, Research Institute of Tsinghua University in Shenzhen, China. HaCaT cells were cultivated in DMEM (REF# C11995500BT, Gibco) with 10% FBS (REF# 10091148, Gibco) and 1% antibiotics (penicillin and streptomycin, REF# 15140122, Gibco) at 37 °C in an incubator with 5% CO_2_. Once 80–90% confluency was reached, hASCs were passaged and the cells with good growth and normal morphology were selected for the follow-up experiments.

### Characterization of hASCs

Based on flow cytometry analysis, the positive expression of CD90 and CD44 and negative or low expression of CD11b, CD34, CD45, and HLADR markers were used as the characterization criteria [46] for hASCs. A total of 10^4^ cells in passage 3 was resuspended in cold phosphate-buffered saline (PBS). After being blocked, the cells’ incubation with PE-coupled antibodies or fluorescein isothiocyanate (FITC) in the darkness was performed for CD90, CD44, CD11b, CD34, CD45, HLADR, and control isotype IgG on ice for 30 min. The analysis was performed in a Guava flow cytometer (Merck Millipore, Billerica, MA). And the results were expressed as the percentage of labeled cells among all analyzed events.

hASCs at P3 (with a density of 5000 cells/cm^2^) were cultured in an osteogenic-specific differentiation medium for 3 weeks. The osteogenic medium contained 50 μM ascorbic acid, 10 M dexamethasone, and 10 mM β-glycerol phosphate. Alizarin Red staining dye was used to detect the differentiated cells to osteoblasts.

### Fabrication of CSSs

Similar to the commercially available bilayer artificial dermis, the CSS (without a silicone layer) involved in this study is also mainly consisted of type I collagen derived from bovine tendon, provided by Lando Biomaterials R&D Center, China. Briefly, with the addition of chondroitin sulfate, the collagen solution was prepared with a final mass fraction of 0.6%. After the mixtures were poured into individual molds and dried in a freeze dryer, a 105 °C thermal crosslinking process was performed under vacuum. The freeze-dried samples were then immersed in glutaraldehyde solution with an optimal concentration (0.25%, w/w), followed by a 24 h crosslinking process at 4 °C. Finally, porous collagen-based CSS was obtained after repeated lyophilization. Scanning electron microscope (SEM; TM3030, HITACHI) was applied to observe the microstructure with Lando® Artificial Dermal Regeneration Matrix products as a control.

### 3D differentiation of hASCs into keratinocytes

**Fig. 1:**
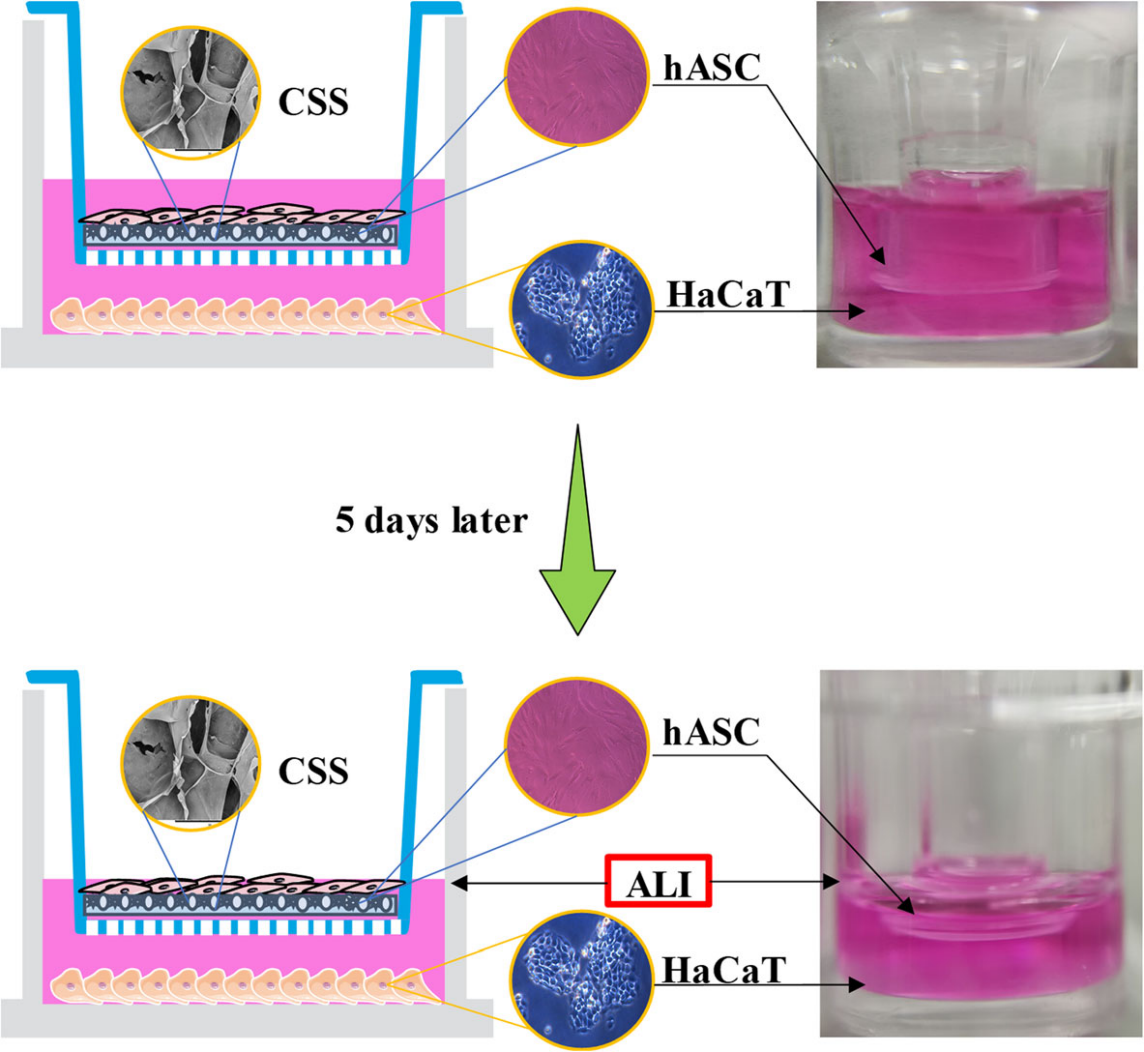
Schematic representation of 3D differentiation of hASCs in keratinocytes via an indirect co-culture strategy

The 3D differentiation strategy is shown schematically in Fig. 1. Before in vitro studies, CSSs (a diameter of 6 mm with a biopsy punch) sterilized by irradiation were immersed in complete DMEM for overnight in a CO_2_ incubator. Based on an indirect co-culture principle, 6.5-mm inserts (0.4 μm pore polyester membrane, REF# 3470, Corning) were placed into 24-well plates (REF# 3524, Corning) pre-seeded with HaCaT cells (over 70% confluency) in 600 μL of complete DMEM. After the CSSs were then transferred onto these inserts, re-suspended hASCs in 100 μL of complete DMEM were plated at a density of 5 × 10^4^ cells/ml over the surface of CSSs. At 100% confluency of HaCaT cells in the bottom of the well, the inserts would be transferred to new 24-well plates containing pre-seeded HaCaT cells.

Five days later, proper culture media was removed from the wells so that the hASCs were cultured at ALI (Fig. 1) which better mimics the skin’s epidermal layer [47], encouraging cells to differentiate and stratify to keratinocytes. In this way, the 3D differentiation model was constructed, and CSSs on the inserts were collected after scheduled intervals (10 and 15 days) for the following evaluations. hASCs cultivated alone on CSSs (without HaCaT cells) were used as control for the co-culture experiment, and hASCs cultivated solely on the polyester membrane of insert (without any CSS, 2D differentiation) were designed as a comparison of the 3D differentiation cultivation. In addition, hASCs co-cultured with HaCaT cells were also immersed in complete DMEM to investigate the impact of ALI on the differentiation.

### Microscopic observation by invert microscope

At different time intervals (10 and 15 days), hASCs undergoing the 3D and 2D differentiation were trypsinized using 0.25% trypsin/EDTA (REF# 25200056, Gibco) and resuspended in new 24-well plates, respectively. Cells attached in culture flasks were observed 2 days later and the changes in cellular morphology were recorded using an invert microscope (CKX31, Olympus).

### Micromorphology via SEM

Based on the existing protocol [48], the surface of the differentiated hASCs on the polyester membrane and cell-seeded CSSs was also observed via SEM. After the culture medium was decanted, the samples were rinsed with PBS twice, fixed in 2.5% glutaraldehyde for 2 h, followed by deionized water to rinse again. The samples were then subjected to a medium gradient dehydration with 50%, 70%, 80%, 90%, 100%, and 100% ethanol for 15 min, respectively. After lyophilization in a freeze dryer overnight, each sample was mounted on an aluminum sample holder and coated with a thin layer of gold under vacuum using a sputtering machine (JS-17085, KYKY) at 1 kV and 5 mA for 60 s. Gold-coated samples were visualized by means of SEM (TM3030, HITACHI) at an acceleration voltage of 20 kV.

### Adhesion and proliferation assay

At intervals, CSSs containing cells were transferred into 96-well plates and gently rinsed twice with PBS (pre-warmed at 37 °C), followed by 4% paraformaldehyde to be fixed for 20 min. After washing the fixative with PBS, the cells were penetrated using 0.1% Triton X-100 (REF# V900502, Sigma) for 10 min and then blocked with 1% bovine serum albumin (BSA; REF# V900933, Sigma) for 30 min. Afterwards, 5 μg rhodamine-labeled phalloidin (REF# P1951, Sigma) in 1 ml PBS was used to stain the cytoskeletons; 4′,6-diamidino-2-phenylindole (DAPI; REF# D9542, Sigma) solution was used to stain the cell nuclei. After two washing steps, cell adhesion and growth conditions on the CSSs were detected by a laser scanning confocal microscopy (LSCM, Leica, SP8) at 405/552 nm. Five random views of each sample were recorded at the same magnifications.

### Hematoxylin and eosin (H&E) staining

All fixed CSSs were followed by dehydration through a graded series of ethanol wash and then embedded in paraffin. After dewaxation and rehydration, sample sections with 10 μm thickness were used for H&E staining following the manufacturer’s (Sigma) standardized protocols. Sections in each group for each time point were visualized using an automatic section scanner (C10730-12, HAMAMATSU), and five random views in each section were selected.

### Immunostaining assay

To assay the expression of a specific marker in keratinocytes and in vitro differentiated hASCs, immunofluorescence staining was performed following the established method [49]. Similar to the adhesion assay, the samples (polyester membranes and CSSs) with cells in 96-well plates (REF# 3599, Corning) also underwent these processes such as being rinsed, fixed, and penetrated. After being blocked with 1% BSA at room temperature for 1 h, the samples were incubated with primary anti-K14 (mouse monoclonal; 1:100, REF# 7800, Abcam) at 4 °C overnight, followed by secondary antibody including FITC-conjugated goat anti-rabbit IgG (mouse monoclonal; 1:100, REF# 6785, Abcam) solution for 1 h at room temperature in a dark place. Nuclei were labeled by DAPI for 5 min and stained samples were kept at 4 °C after two washing steps. The cells were inspected and photographed using a laser scanning confocal microscopy (LSCM; SP8, Leica) at 493/528 nm. The average fluorescence intensity per unit area was calculated using Image-Pro Plus software (version 6.0). Five low-magnification (× 20) images of the individual samples in each group were used for calculation.

### Quantitative real-time polymerase chain reaction (qPCR)

qPCR was performed to analyze the expression of K14 (one of the keratinocyte markers), OCT-4 (one of stem cell markers), and MET (mesenchymal-epithelial transition) specific genes (E-cadherin and N-cadherin). Briefly, total RNA was extracted from the samples using a TRIzol Reagent (REF# 15596026, Invitrogen) according to the manufacturer’s instructions. Subsequently, RNA was reverse transcribed into cDNAs using the cDNA synthesis kit (REF# AB1453B, Thermo Scientific). The cDNAs were amplified through qPCR by SYBR Green Real-Time PCR Master Mix (REF# QPK-201B, TOYOBO). The relative expression of candidate genes was analyzed using the 2−ΔΔCt method and normalized to GAPDH. All the specific primers were shown in Table 1.

**Table 1:** Sequences of primers used for qPCR

Gene name	Direction	Primer sequence (5′-3′)
K14	Forward	GCAGTCATCCAGAGATGTGACC
	Reverse	GGGATCTTCCAGTGGGATCT
OCT-4	Forward	ACCCCTGGTGCCGTGAA
	Reverse	GGCTGAATACCTTCCCAAATA
E-cadherin	Forward	GGTGCTCTTCCAGGAACCTC
	Reverse	GGAAACTCTCTCGGTCCAGC
N-cadherin	Forward	GTACAGTGTAACTGGGCCAGG
	Reverse	GATCCAAGTCCAGCTGCCACTG
GAPDH	Forward	ACCCACTCCTCCACCTTTGA
	Reverse	ACGAATTTGGCTACAGCAACAG

### Statistical analysis

All data are presented as mean values ± SD (standard deviation) from at least three independent experiments. Statistical analyses were performed using SPSS software (version 23.0, IBM Corporation). The unpaired Student’s *t* test was used to analyze the differences between the two groups. One-way analysis of variance was used to analyze differences among three or more groups. Differences were considered statistically significant for *p* < 0.05 (*), *p* < 0.01 (**), and *p* < 0.001 (***), and ns indicates not significant.

## Results

### Characterization of hASCs

**Fig. 2:**
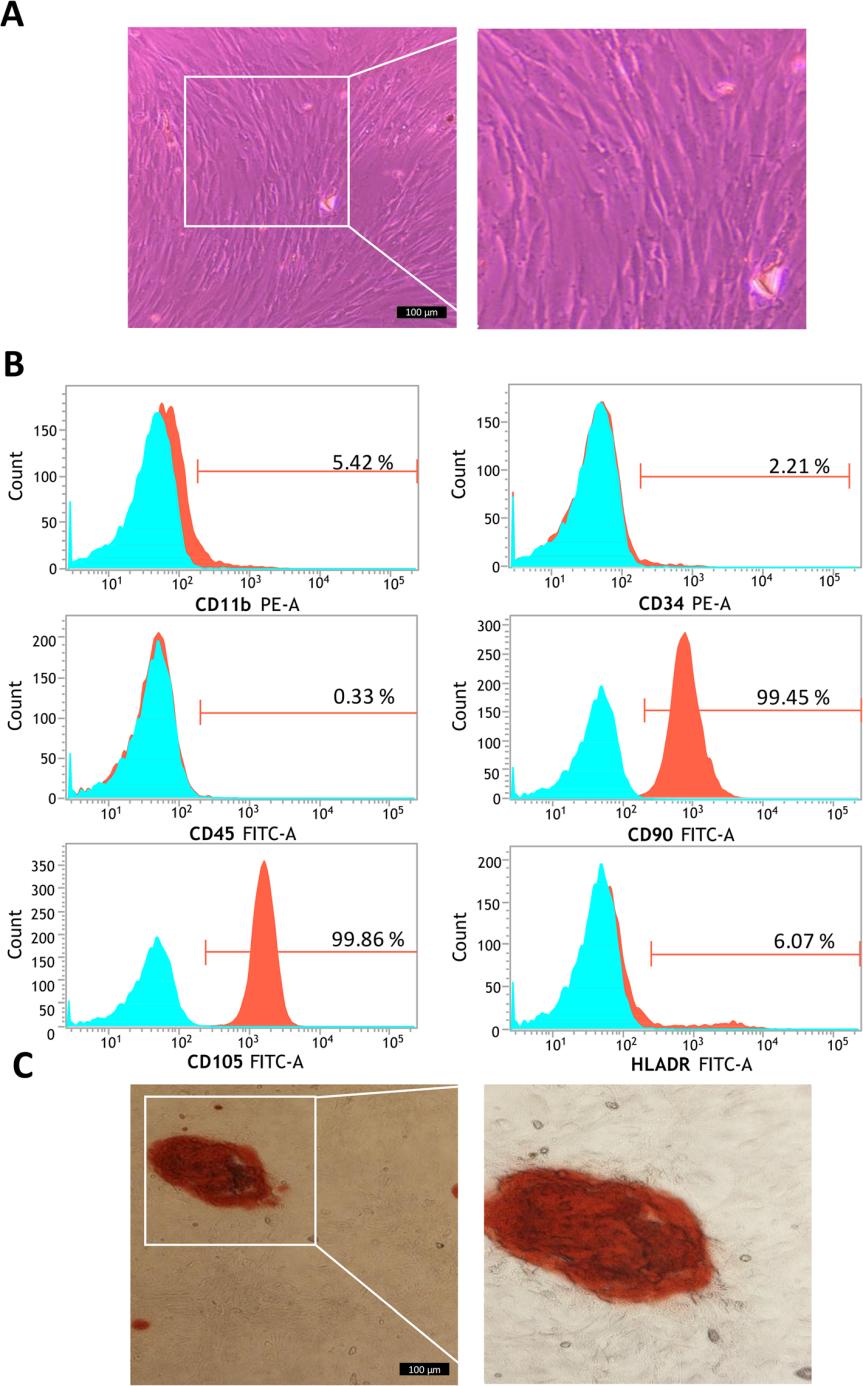
Characterization of hASCs by cellular morphology, immunophenotyping and differentiation potential assay. **a** Typical morphology of hASCs. **b** Immunophenotyping of hASCs (P3), histograms indicate the positive mean value of each marker. **c** Mineralization following osteogenic differentiation as visualized by Alizarin Red staining

Frozen hASCs of P3 successfully adhered to petri dishes after recovery and reached > 90% confluency when cultured in complete DMEM 3 days later. Cells were found to be spindle-like in morphology and had the potential to be arranged in a whirlpool as shown in Fig. 2a. According to the flow cytometry analysis, the hASCs were shown to be positive for cell surface marker CD90 and CD44, but a negative or low expression for CD11b, CD34, CD45, and HLADR markers (Fig. 2b). Alizarin Red staining after cultivation with osteogenic medium showed differentiation into osteoblasts with their typical matrix mineralization (Fig. 2c). Therefore, the cells were characterized as human MSCs [50] which were utilized for the following experiments.

### CSSs desired were successfully fabricated

**Fig. 3:**
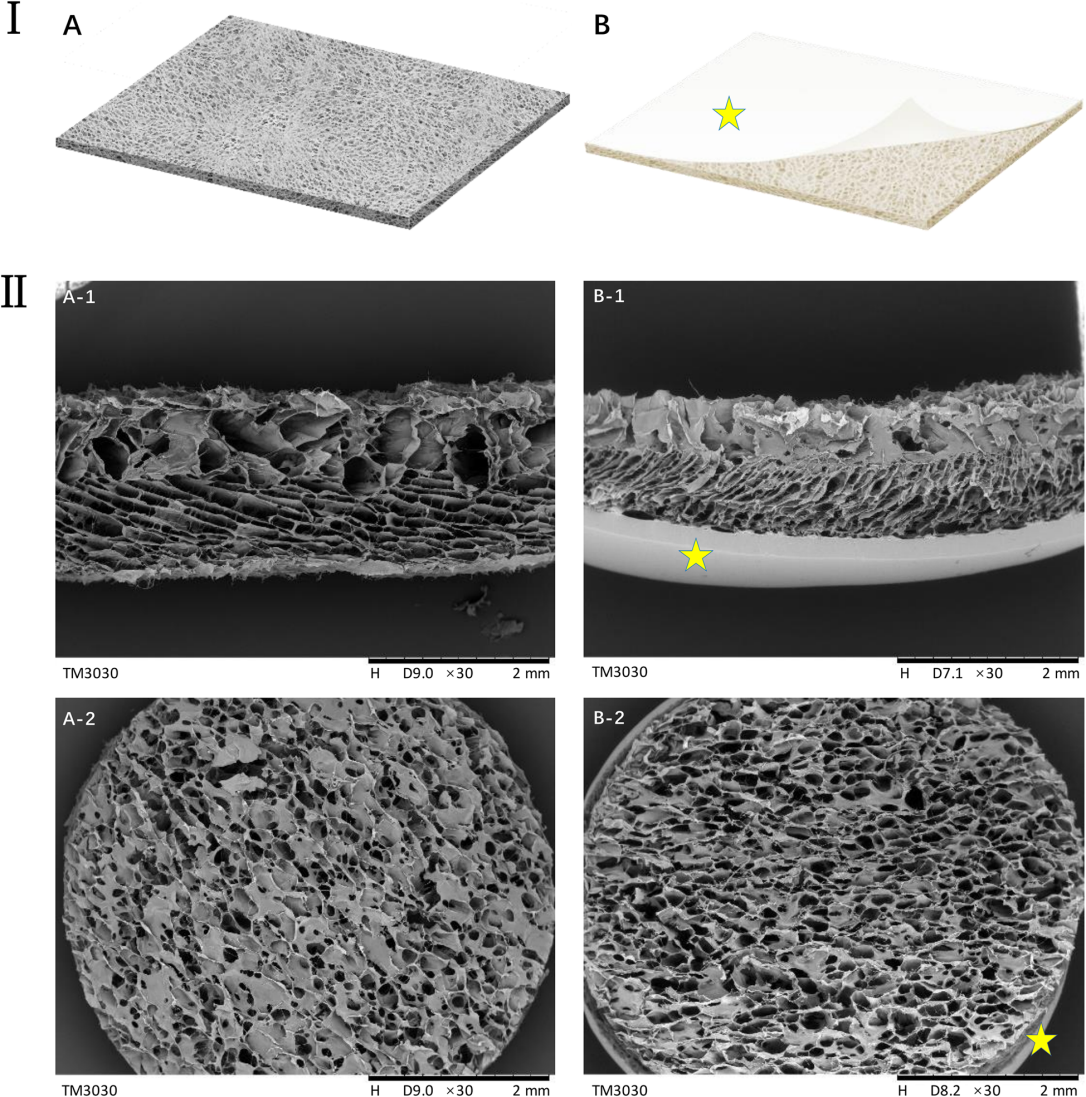
Comparisons between CSSs fabricated and the Lando® Artificial Dermal Regeneration Matrix products. **I** Schematic diagram of the scalffolds. **II** SEM images of the scalffolds ((A) The CSSs fabricated; (B) the Lando® Artificial Dermal Regeneration Matrix products, yellow pentagram indicates the silicone layer; (A-1) side view; (A-2) surface view)

Unlike artificial dermis commercially available, the CSS in this research was a monolayer structure mainly containing collagen and polysaccharides without a silicone layer (Fig. 3a). SEM micrographs (Fig. 3b) show that these fabricated CSSs presented a porous structure with pore sizes ranging from 50 to 150 μm and a continuous radial structure, which is beneficial for nutrient transport, cell migration, and angiogenesis [48]. There was no significant difference between the CSSs and Lando® Artificial Dermal Regeneration Matrix products.

### Microscopic observation of differentiated hASCs

**Fig. 4:**
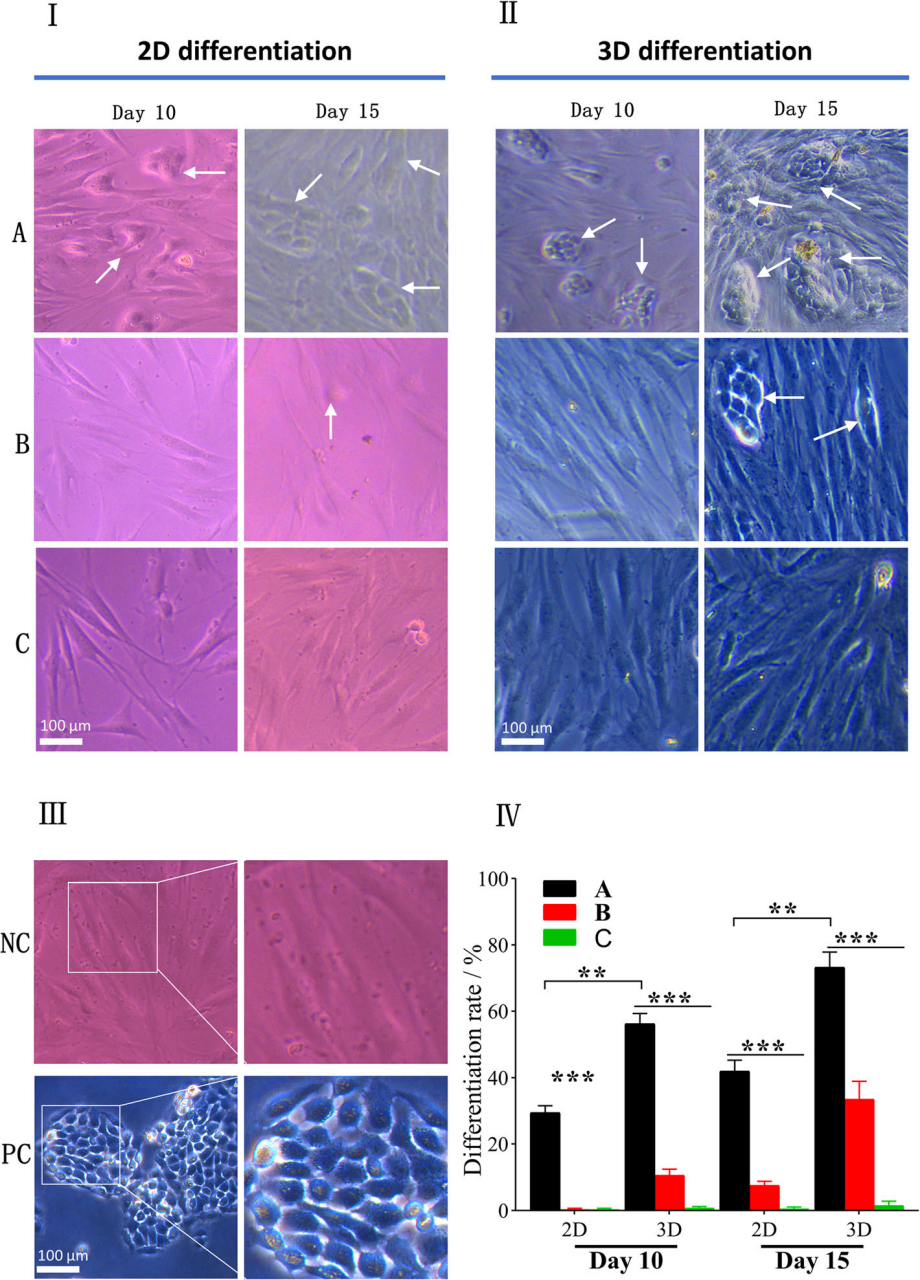
Morphology of differentiated hASCs under invert microscope. hASCs with typical cobblestone morphology similar to HaCaT cells were considered differentiated cells (white arrow). **I** 2D differentiation ((A) hASCs indirectly co-cultured with HaCaT cells at ALI; (B) hASCs indirectly co-cultured with HaCaT cells under ALI; (C) hASCs cultured under ALI without HaCaT’s co-cultivation). **II** 3D differentiation. **III** Controls ((NC) undifferentiated hASCs as negative control, (PC) HaCaT cells as positive control). **IV** Quantification of differentiation rate (%) at different time points (**p < 0.01, ***p < 0.001)

hASCs undergoing the 3D and 2D differentiation successfully attached to the bottom of new 24-well plates after 2 days of their seeding. It was observed that after 10 days of culture, only hASCs in co-culture with HaCaT cells at ALI began to clustering and exhibited typical cobblestone morphology similar to HaCaT cells (as a positive control) (Fig. 4II). At day 15, cobblestone-like differentiated cells were all observed in the respective plate wells via co-cultivation with HaCaT cells, of which the hASC-derived keratinocytes undergoing 2D differentiation were remarkably less than those cultured on CSSs (*P* < 0.01, Fig. 4). In contrast, hASCs without the co-cultured HaCaT cells did not show any significant morphological changes, whether they were pre-cultivated on the polyester membranes or on the CSSs. The differences in morphology and quantification of differentiation among hASCs in each group are shown in Fig. 4IV.

**Fig. 5:**
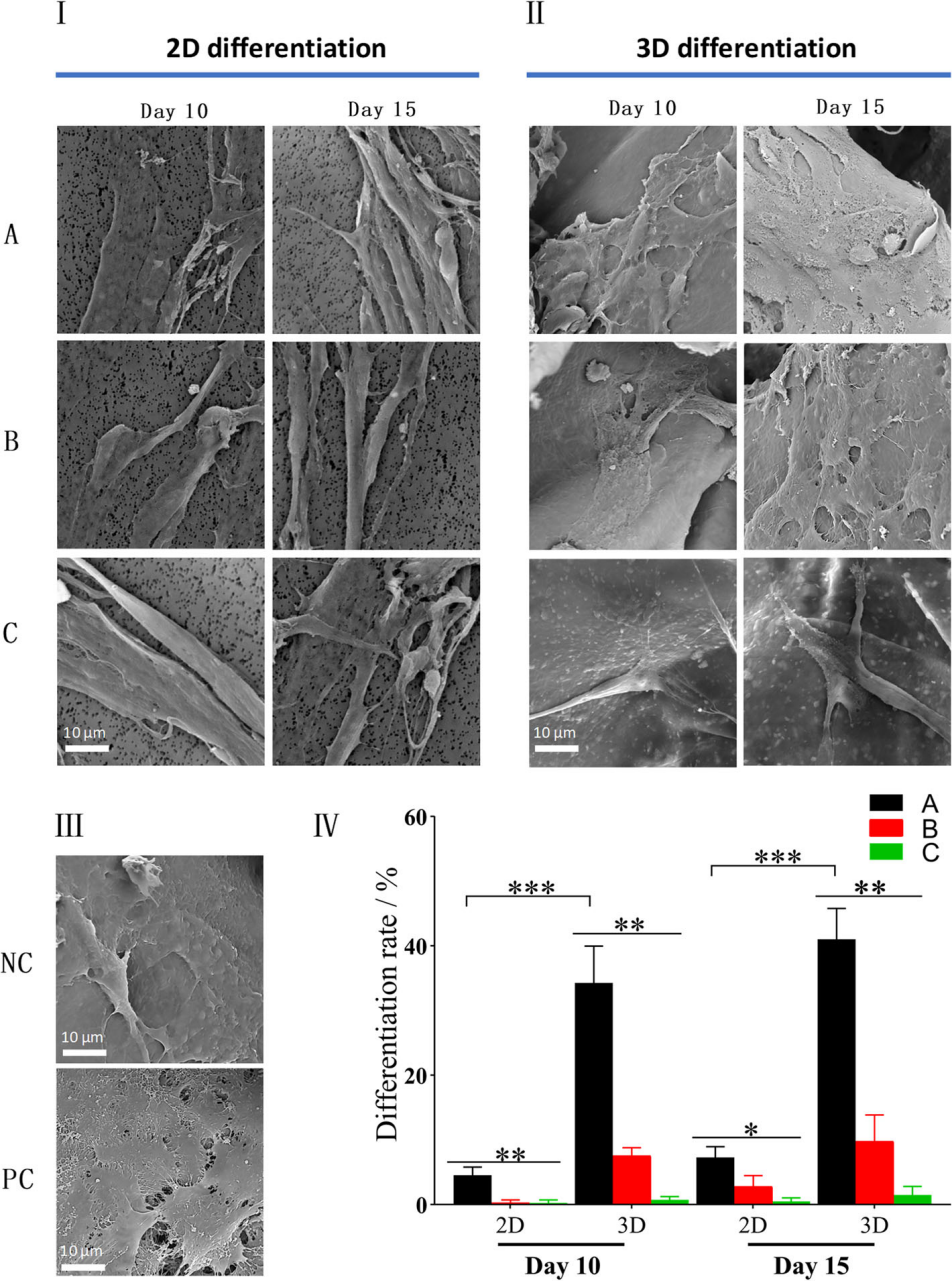
SEM analysis of differentiated hASCs in each group on day 10 and 15. **I** 2D differentiation ((A) hASCs indirectly co-cultured with HaCaT cells at ALI; (B) hASCs indirectly co-cultured with HaCaT cells under ALI, (c) hASCs cultured under ALI without HaCaT’s co-cultivation). **II** 3D differentiation. **III** Controls ((NC) undifferentiated hASCs as negative control, (PC) HaCaT cells as positive control). **IV** Quantification of differentiation rate (%) at different time points (*p < 0.05, **p < 0.01, ***p < 0.001)

Unlike cells observed under the inverted microscope that need to be pre-seeded on new plates in advance, the samples containing hASCs can be directly processed by SEM after pretreatment. The SEM images revealed that the hASCs in each group fully spread on CSSs and polyester membranes (Fig. 5). In line with the phenomenon observed under the invert microscope, hASCs co-cultured with HaCaT cells via 3D differentiation showed more colonies of keratinocyte-like cells on day 10 (*p* < 0.001) and day 15 (*p* < 0.001), which were distinguished from those under 2D differentiation or without co-cultivation.

### Adhesion and proliferation of hASC on CSSs

**Fig. 6:**
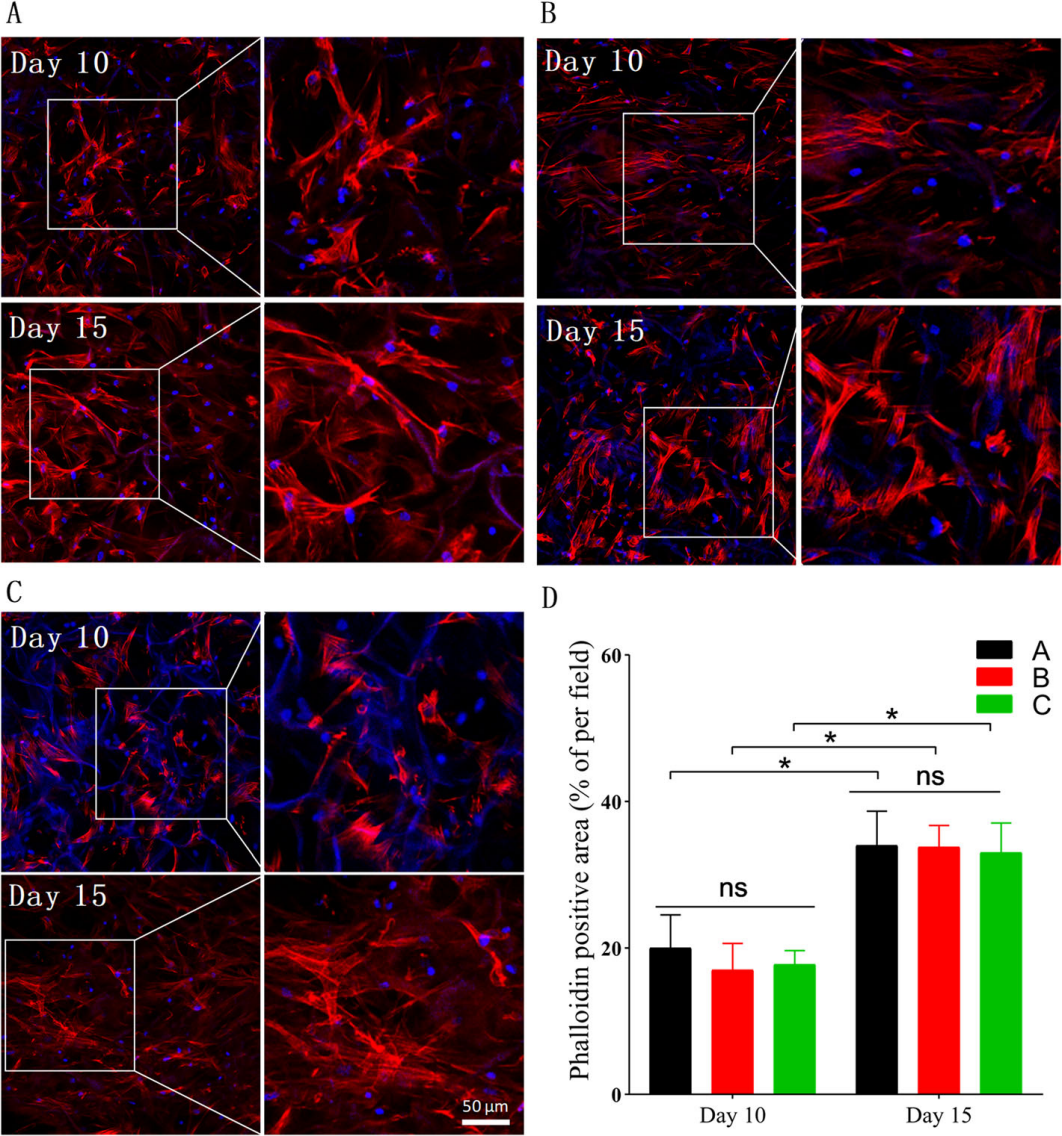
LSCM images of hASCs adherence and proliferation onto the CSSs after 10 and 15 days of cultivation. **a** hASCs indirectly co-cultured with HaCaT cells at ALI. **b** hASCs indirectly co-cultured with HaCaT cells under ALI. **c** hASCs cultured under ALI without HaCaT’s co-cultivation. **d** Quantification of phalloidin positive area (%) at different intervals (*p < 0.05)

At intervals (day 10 and day 15), CSSs containing cells were chosen for observing cell adhesion and growth condition by staining with Alexa Fluor 564 Phalloidin for cytoskeleton and DAPI for the nucleus. Figure 6 shows that after 10 days of culture, hASCs had firmly attached to the pore walls of CSSs in each group and had spread with good proliferation. After 15 days of culture, more cells had fully spread and proliferated inside the pores along the pore walls, which indicated good cell adhesion and proliferation. And as shown in Fig. 6d, the fluorescence intensity confirmed that there was no significant difference among the cells in each 3D differentiation group at the same interval (*p* > 0.05).

**Fig. 7:**
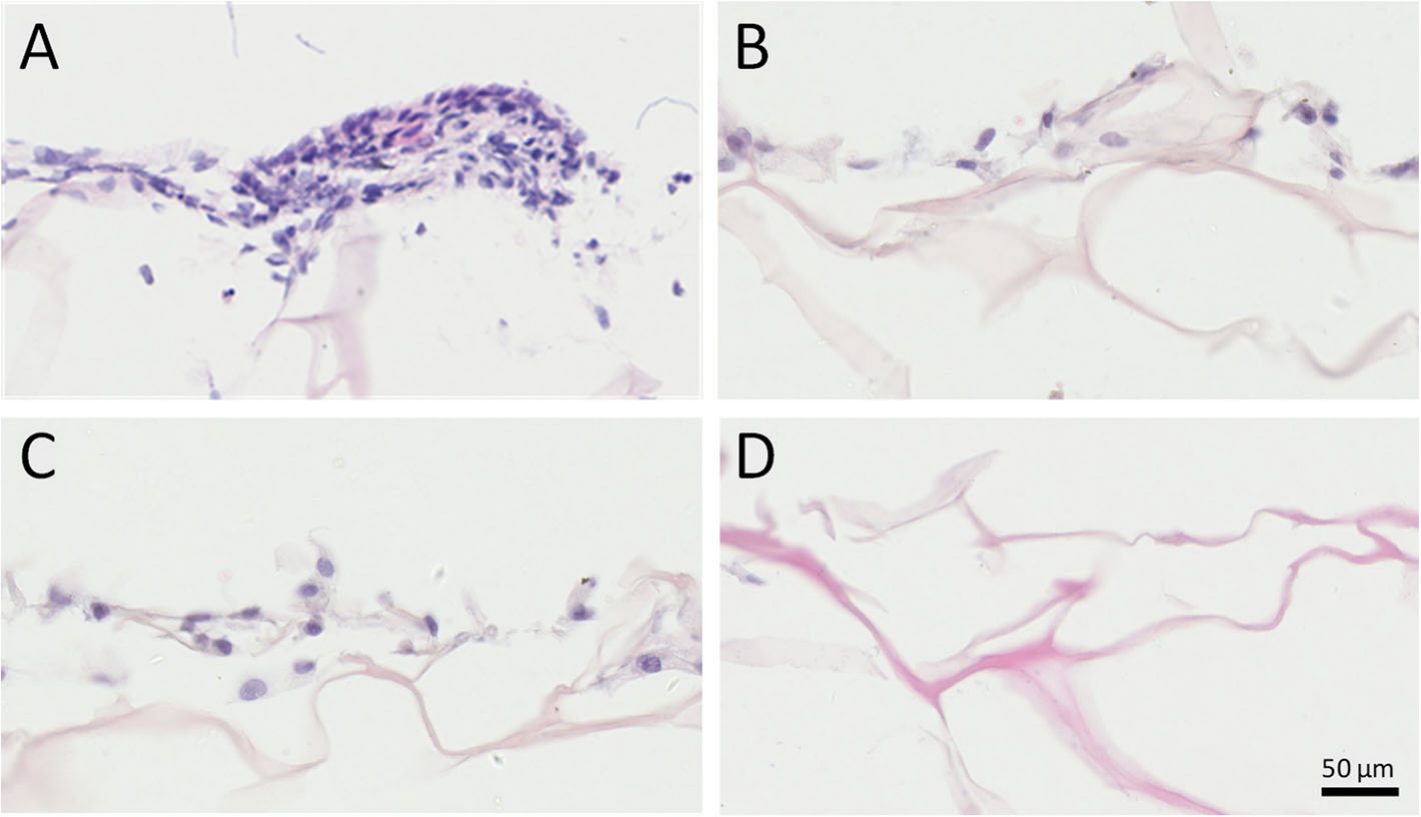
H&E staining of histological sections of cells differentiated on CSSs on day 15. **a** hASCs indirectly co-cultured with HaCaT cells at ALI, exhibiting more colonies and closely packed together. **b** hASCs indirectly co-cultured with HaCaT cells under ALI (immersed in culture medium). **c** hASCs cultured under ALI without HaCaT’s co-cultivation. **d** CSSs fabricated without any hASCs seeded on (as blank control)

After 15 days of culture, sample sections of CSSs obtained were processed by H&E staining for further morphological analysis. Desired cellular growth was observed on and inside the CSSs in all groups (Fig. 7), which was consistent with the adhesion assay. Interestingly, H&E staining also revealed that hASCs co-cultured with HaCaT cells at ALI exhibited more colonies and closely packed together (Fig. 7a), showing more similar to primary keratinocytes than undifferentiated hASCs with long spindles and elongated nuclei.

### Immunofluorescence staining of keratinocytes marker

**Fig. 8:**
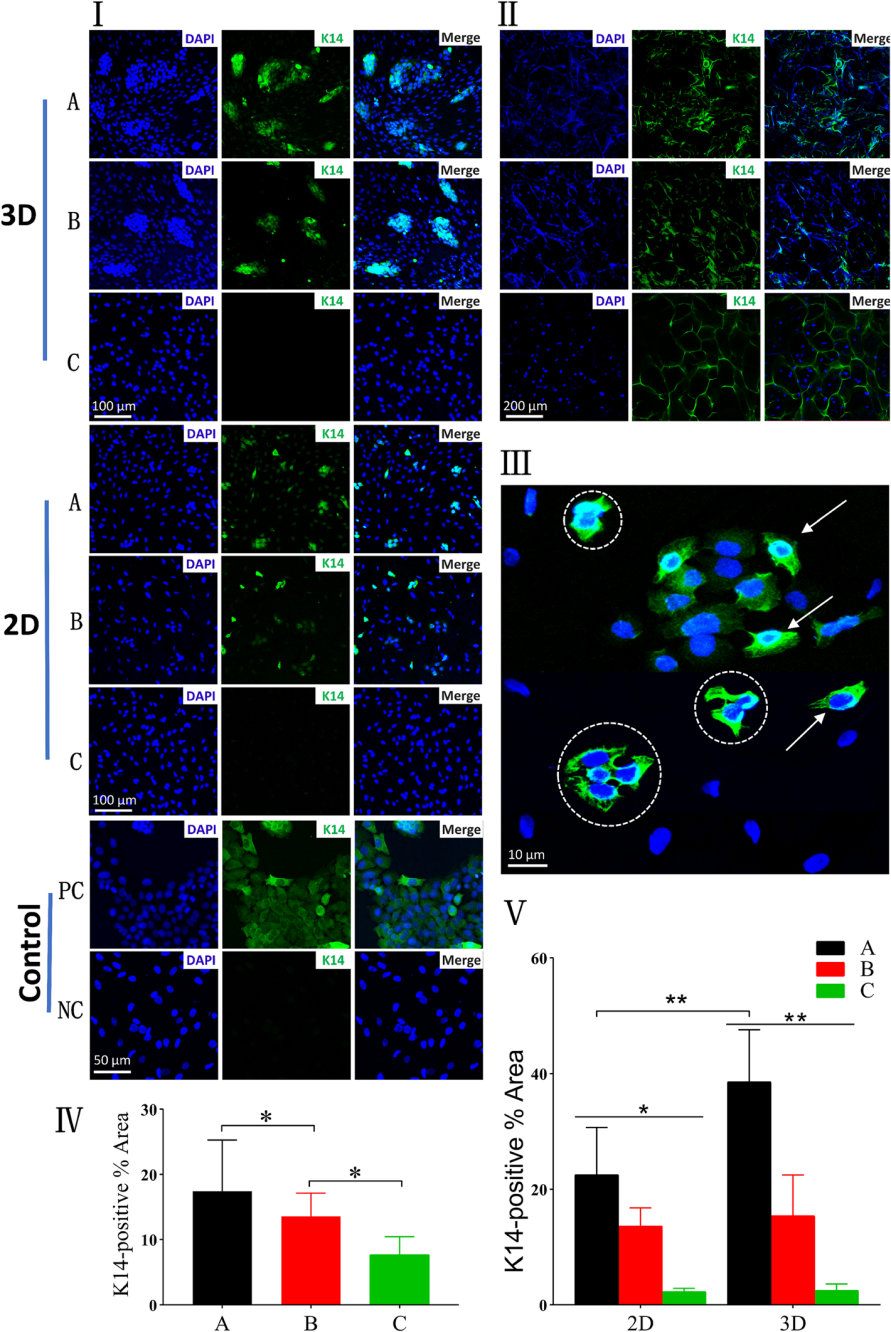
Protein-expression analysis of K14 evaluated by immunofluorescence staining on day 15. Similar to HaCaT cells, hASCs-derived keratinocytes (differentiated hASCs) showed positive staining of K14 (green). Nucleus were stained with DAPI (blue). **I** Immunofluorescence staining images of glass slides seeded hASCs in each group. **II** Immunofluorescence staining images of CSSs containing hASCs (3D differentiation). **III** Typical image of glass slides seeded hASCs trypsinized from CSSs co-cultured at ALI, polygonal cells with keratinocyte-like morphology (white arrow), and multinucleated fused cells (white dotted circle) were observed. **IV** Quantification of K14 positive area (%) of CSSs containing hASCs, corresponding to **II**. **V** Quantification of K14 positive area (%) of glass slides seeded hASCs, corresponding to **I** ((A) hASCs indirectly co-cultured with HaCaT cells at ALI, (B) hASCs indirectly co-cultured with HaCaT cells under ALI, (C) hASCs cultured under ALI without HaCaT’s co-cultivation, (PC) HaCaT cells as positive control, (NC) undifferentiated hASCs as negative control) (*p < 0.05, **p < 0.01)

Despite the above morphology observations might suggest differentiation of hASCs towards keratinocytes, more in-depth assays were necessary to confirm the epithelial lineage. At the end of the induction period (day 15), immunostaining assay was done to detect the expression of K14 at the protein level. The upregulation level of K14 (indirectly assessed by green fluorescence intensity) was seen in the CSSs in co-culture with HaCaT cells at ALI than others (Fig. 8 II, IV). But for all the scaffolds, high autofluorescence was inevitably observed, which seriously interfered with the fluorescence emitted by differentiated hASCs.

To address this limitation, hASCs cultured on the polyester membrane of inserts and CSSs were trypsinized and cytospinned onto glass slides coated with poly-lysine (10^4^ cells/slide). Immunostained cell slides demonstrated that both hASCs co-cultured with HaCaT cells on 3D and 2D culture system could express K14 (Fig. 8I, A and B), of which the expression intensity was higher in differentiated hASCs in 3D compared to 2D co-culture system (*p* < 0.01, Fig. 8V), accompanied by keratinocyte-like morphology and more colonies (Fig. 8I). What is more, it was observed that some differentiated hASCs retained a single nucleus whereas others possessed multiple nucleus, of which the possible reason is that cellular fusion functions in the transdifferentiation of adult stem cells towards epithelial lineages [32]. However, the expression of this marker in both systems was less than HaCaT cells (Fig. 8 I, PC). And hASCs cultivated without co-culture of HaCaT cells did not show remarkable expression of K14 (Fig. 8I, C).

### Gene expression analysis

**Fig. 9:**
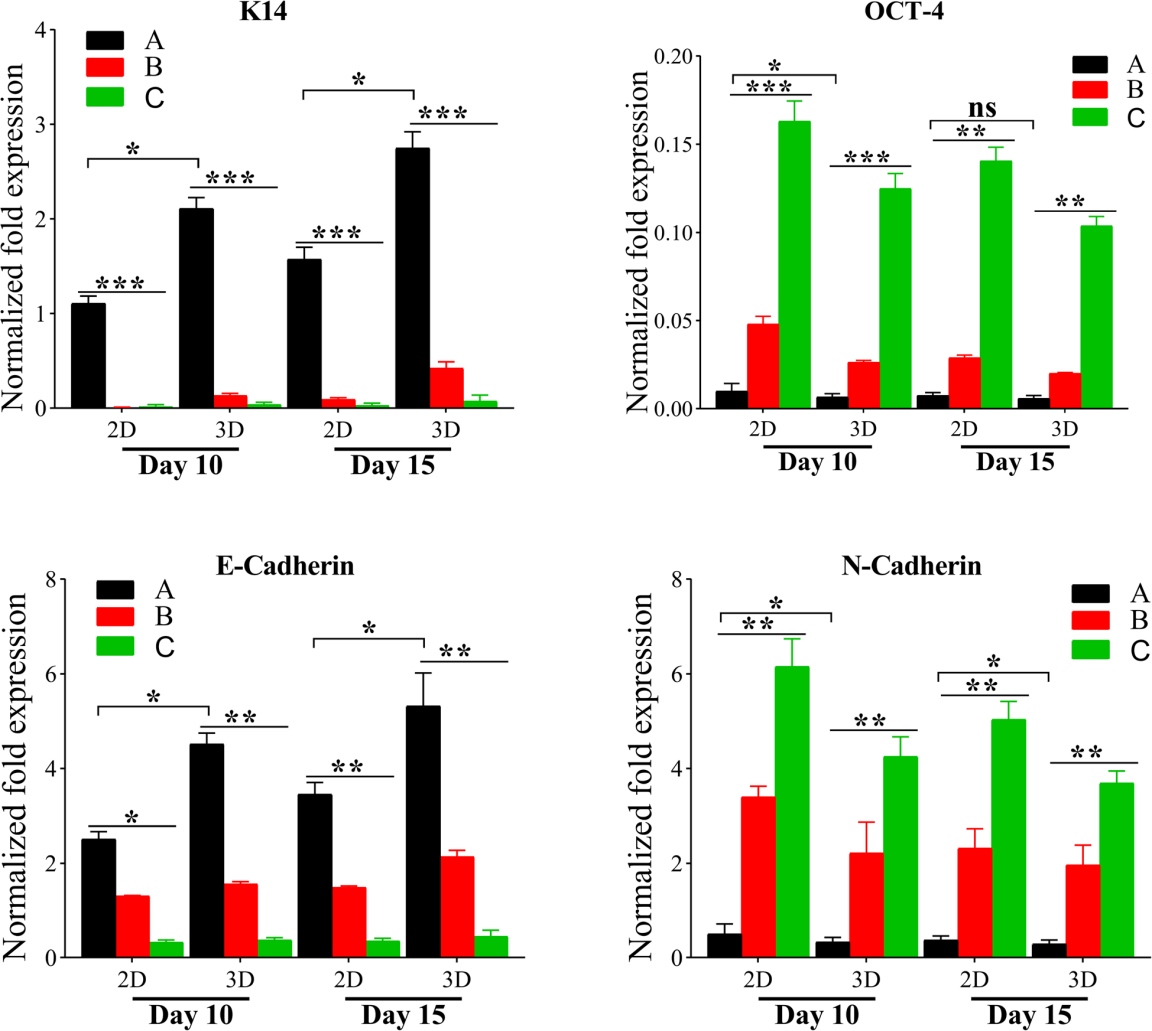
Gene-expression analysis of K14, OCT-4, E-cadherin, and N-cadherin evaluated by qPCR on day 10 and 15. (A) hASCs indirectly co-cultured with HaCaT cells at ALI. (B) hASCs indirectly co-cultured with HaCaT cells under ALI (immersed in culture medium). (C) hASCs cultured under ALI without HaCaT’s co-cultivation (*p < 0.05, **p < 0.01, ***p < 0.001)

The synthesis of K14 was examined at the gene expression level with qRT-PCR, which were normalized to corresponding GAPDH. Compared to 2D differentiation system, hASCs co-cultured on CSSs showed a significant increase of K14 mRNA expression on both day 10 (*p* < 0.05) and day 15 (*p* < 0.05), as exhibited from Fig. 9. hASCs without co-cultivation or via submerged culture showed low or almost no K14 expression, consistent with the immunostaining assay.

Apart from the detection of K14 expression level to prove the differentiation of hASCs into the epithelial lineage, in turn, it is necessary to detect OCT-4 expression to verify whether the stemness is downregulated. The mRNA level of OCT-4 in hASCs co-cultured on CSSs was significantly lower than others (Fig. 9), which seems to be negatively related to the degree of hASC differentiation. It was worth noting that with the increase of culture time, OCT-4 expression of these hASCs without significant differentiation also decreased. This might be related to the susceptibility of MSCs to aging as the number of passages increases [51], which might be why P3–P5 of hASCs are often proposed for experiments.

MET is fundamental evolutionarily conserved mechanisms for cell fate conversion [52, 53], accompanied by downregulated mesenchymal genes (E-cadherin, ZEB1, etc.) and upregulated epithelial genes (E-cadherin, P63, etc.) [54]. In line with the degree of hASC differentiation, the mRNA expression levels of E-cadherin in differentiated cells were higher than those with low or no significant differentiation (Fig. 9). In contrast, the N-cadherin expression level of differentiated hASCs was lower than others (Fig. 9).

## Discussion

Treatment of various skin wounds, accompanied by existing clinical challenges (like increasing morbidity, and rising risk of amputations) [1, 3], is still a challenging task in clinical practice [4, 5]. Wound healing with four-phase process requires the joint participation of various cell types, of which keratinocytes (the main component of the skin’s epidermis) play an important role in promoting re-epithelialization for wound closure and barrier repair [47]. However, keratinocytes require specific culture conditions and easily become senescent after only 10–20 doublings, which hampers the production of sufficient cells for graft [16].

With advances in cell therapy and regenerative medicine, MSCs have been proposed as promising candidates towards epithelial differentiation [55]. The main strategy for the differentiation of stem cells into keratinocytes is to add specific culture supplements [22, 23], by conditioned medium from keratinocytes or co-cultivation (contact or indirect) with keratinocytes [29]. Due to better mimic of the skin environment, some researchers reported that ASCs lifted to ALI showed a satisfactory differentiation [16]. Otherwise, differentiation of stem cells depends on the “niche,” including diffusible paracrine effects, ECM, and cellular and mechanical factors [21, 26, 27, 56], and most studies are based solely on 2D induction culture missing the biophysical microenvironments of the ECM. In contrast, 3D system using scaffolds (with interconnected pores) which mimic the structure and function of ECM proteins not only promotes cell adhesion, cell-biomaterial interactions, and cell proliferation [10, 38, 39], but also facilitates differentiation in skin regeneration [10, 57, 58].

Over the past years, major efforts have been done to produce biomaterial scaffolds suitable for tissue engineering, of which the main raw materials include (but not limited to) gelatin-hyaluronan, collagen, chitosan, etc. [32, 46, 59–61] The typical one is bilayer artificial dermis commercially available, mainly composed of type I collagen, which are widely applied for scar-free wound healing [6]. However, it was unclear whether the 3D culture of ASCs on artificial dermis can be better transdifferentiated into keratinocytes and even whether different culture conditions would give birth to diverse effects. The monolayer CSSs similar to the collagen layer of bilayer artificial dermis commercially available were utilized currently.

In this study, hASCs (a type of MSCs) were selected due to the advantages (like wide sources, convenient access strategies, and less damage to patients [1, 20]). Based on the spindle-like cellular morphology, flow cytometry analysis, and osteogenic differentiation, hASCs of P3 were characterized as human MSCs. And SEM demonstrated that there was no significant difference between the CSSs fabricated and bilayer artificial dermis commercially available (Fig. 3). According to the hypothesis that hASCs on 3D system co-cultured with keratinocytes at ALI would better transdifferentiate into epithelial lineages, HaCaT cells were cultured on the bottom of the plate wells to create an indirect co-cultivation by means of Transwell inserts. As a comparison (2D differentiation), hASCs were co-cultured on the polyester membranes, and those without co-culture and under ALI were as a control.

Under the invert microscope, phenotypic and morphological characteristics of human keratinocytes as a polygonal cell with a cobblestone pattern [62] were only observed in hASCs co-cultured with HaCaT cells at ALI, and hASC-derived keratinocytes undergoing 2D differentiation (on day 15) were remarkably less than those differentiated on CSSs (*p* < 0.01, Fig. 4), which was in line with the SEM images (Fig. 5). The adhesion assay confirmed that there was no significant difference among the cells in each 3D differentiation group at the same interval (*p* > 0.05, Fig. 6), indicating the diverse culture strategies had little interface with the adhesion and proliferation of hASCs, which was fundamental for the following quantification. It is weird that each 3D differentiation group showed good proliferation with no significant difference at the same interval, which differed from the consensus that early strong differentiation signals may inhibit the proliferation process [63]. Whether this phenomenon was related to the application of 3D scaffolds remained unclear. Further analysis via HE staining not only demonstrated desired cellular growth on and inside the CSSs in all groups (in line with the adhesion assay) (Fig. 7), but also interestingly revealed that hASCs co-cultured with HaCaT cells at ALI exhibited more colonies and closely packed together (consistent with microscopic observation) (Fig. 7a).

Apart from the preliminary assessment via microscopic observation or adhesion assay, the differentiation potential was further confirmed via protein and mRNA expression studies. K14 is an epithelial basal layer marker as described in previous studies [31]. The pluripotency-associated transcription factors, such as OCT-3/4 and SOX-2 [55], are identified as “stemness” genes in undifferentiated human embryonic stem cell lines [55]. In the current study, the significant expression of K14 at the protein (Fig. 8) and obvious upregulation in the mRNA level (Fig. 9) could be found in the CSSs undergoing 3D co-cultivation at ALI on day 15. Notably, high autofluorescence was inevitably observed in all the scaffolds, which seriously interfered with the fluorescence emitted by differentiated hASCs, and the autofluorescent nature of collagen may be responsible for this phenomenon [32, 64, 65]. Accordingly, glass slides were created to demonstrate that both hASCs co-cultured with HaCaT cells on 3D and 2D culture system could express K14, of which the expression intensity was higher in the hASCs by 3D differentiation with keratinocyte-like morphology and more colonies compared to 2D co-culture system (*p* < 0.01). Almost in line with the immunofluorescence staining, a significant increase in the mRNA expression of K14 was observed in the 3D co-cultured hASCs with decreased OCT-4 when compared with the others (Fig. 9). In addition, a decreased OCT-4 expression was also observed in those undifferentiated hASCs with the increase of culture time (Fig. 9), and the susceptibility of MSCs to aging with the number of passages increasing might be involved [51], indicating why P3–P5 of hASCs are often proposed for experiments.

To this end, although the results above demonstrated the CSSs better facilitated hASCs differentiation towards epithelial lineages when co-cultured with HaCaT cells at ALI, however, the in-depth mechanisms mediating this special induction pattern in vitro still remained unknown. Epithelial-mesenchymal transition (EMT, widely considered to be associated with tumor metastasis [36]) and its reverse process MET are basic mechanisms for cell fate conversion [52]. MET involves the progressive increase of epithelial cell polarity and upregulation of junctional complexes to form tight junctions at the apex of the lateral domain and the organization of cytoskeletal structures and organelles [53], accompanied by downregulated mesenchymal genes (E-cadherin, ZEB1, etc.) and upregulated epithelial genes (E-cadherin, P63, et c) [54]. In the current research, MET was hypothesized to preliminarily explain the differentiation of hASCs in 3D system at ALI towards keratinocytes. And consistent with the degree of hASC differentiation, the mRNA expression levels of E-cadherin in differentiated cells were higher than those with low or no significant differentiation (Fig. 9). In contrast, the N-cadherin expression level of differentiated hASCs was lower than others (Fig. 9). The results above might initially imply in-depth mechanisms to be associated with MET.

There are actually several limitations in this current research. Firstly, considering that the principle of co-cultivation is mainly by secretion of diffusible specific growth factors and cytokines through a paracrine mechanism [1, 31], new plates were pre-seeded on HaCaT cells to reach over 70% confluency for ensuring that hASCs were always in an appropriate differentiation “niche,” however, causing the operation to be more complex. Secondly, since hASCs were designed to be seeded on CSSs in inserts and in order to avoid the silicone layer’s interface with the paracrine effect, the bilayer artificial dermis was not actually utilized in this experiment, and thus, the results based on the monolayer CSSs failed to completely reflect the commercial artificial dermis. Furthermore, various markers are involved in keratinocytes’ proliferation and migration, with keratin 5 (K15), 14 (K14), and 15 (K15) being used for the basal layer and with keratin 1 (K1) and 10 (K10) for the spinous layer [66] This manuscript provides an early-stage research about the benefits of co-culturing hASCs on collagen sponge scaffolds, and more markers (e.g., K5, K10, and involucrin) and in vivo studies (e.g., pilot experiments in mice model) are needed to verify why it has better differentiation on 3D scaffold to keratinocytes. What is more, although MET was initially implied to elaborate the differentiation pattern of hASCs into keratinocytes, in-depth mechanisms involved in vitro still remained unclear.

## Conclusions

In summary, accumulative data in the study demonstrated again that hASCs had promising potential to differentiate to keratinocytes, which might overcome the limitations with cell therapies to regenerate injured skin in clinical practice. Furthermore, the CSS similar to bilayer artificial dermis commercially available is an in-time, accessible and natural biomaterial which facilitates hASC transdifferentiation into keratinocytes. This in vitro study, although fails to fully reflect the in vivo therapeutic effect, can provide several references for future animal experiments and clinical trials, implying that hASCs seeded on the CSSs may act as an excellent strategy for faster wound healing.

## Data Availability

All data are included in this published article.

## Abbreviations

ECM: Extracellular matrix
(h)ASC(s): (Human) adipose-derived stem cell(s)
MSCs: Mesenchymal stem cells
ALI: Air-liquid interface
K14: Keratin 14
2D: Two-dimensional
3D: Three-dimensional
CSS(s): Collagen sponge scaffold(s)
DMEM: Dulbecco’s modified Eagle’s medium
FBS: Fetal bovine serum
P3: 3rd passage
P5: 5th passage
PBS: Phosphate-buffered saline
FITC: Fluorescein isothiocyanate
SEM: Scanning electron microscope
BSA: Bovine serum albumin
DAPI: 4′,6-Diamidino-2-phenylindole
H&E staining: Hematoxylin and eosin staining
qPCR: Quantitative real-time polymerase chain reaction
MET: Mesenchymal-epithelial transition
SD: Standard deviation
EMT: Epithelial-mesenchymal transition
